# Robust design of a multirotor aerial vehicle

**DOI:** 10.1038/s41598-021-00413-4

**Published:** 2021-10-27

**Authors:** Abhishek Dutta

**Affiliations:** Department of Electrical and Computer Engineering, University of Connecticut, Storrs, 06269 USA

**Keywords:** Electrical and electronic engineering, Aerospace engineering

## Abstract

This paper introduces the methodology of systematic robust design of a multirotor vehicle as an example of how to carry out the robust design of a physical system. Robustness in aerial vehicles is highly desirable as it guarantees a desired level of performance even under environmental uncertainties. Thus far, robustness has been considered in terms of active control in the space of multirotor vehicles, but exploration of the design space itself is lacking. In this work, a conceptual design followed by a robust design is performed to come up with the specifications that lead to least uncertain performance of the multirotor vehicle with respect to stochastic wind disturbances.

## Introduction

The purpose of robust product design is to create a product that is as insensitive to internal and/or external variations as possible. This research explores the design of physical parameters of an aerial vehicle to make it least sensitive to variations in the form of wind disturbances as an example. The result is proof that the design choices discussed are in fact optimally robust. Robust design involves the task of designing a system in which performance variations due to both noise factors and control factors are minimized^1^. Procedures for robust design have been investigated in the literature. For example, Response Surface Methodology was incorporated with the compromise Decision Support Problem^2^. Robust design methods such as the Taguchi design method are widely used and involve evaluating the parameters to be designed, designing the parameters using an orthogonal array, and performing analysis of variance using ANOVA and signal to noise ratios^3^.

In the field of robust aircraft design, the design of fixed wing aircraft using robust methods was explored^4^. The first-order method of moments and Sigma-Point reduced quadrature are used to calculate the mean and variance of the evaluated performance for a fixed wing aircraft^5^. In control theory, robust control deals with designing a controller in the presence of variations or uncertainties either in the system itself or due to external disturbances^6^. Robust control is applied to many dynamical systems and is very popular in flight control systems^7,8^. Aircraft attitude control has been performed with variation in the aircraft model involving weight and center of gravity^9^. Robust control is used in the design of hypersonic aircraft to control longitudinal motion because stability of aircraft at speeds above the speed of sound is very sensitive to environmental conditions^10^. Therefore, a linear quadratic regulator-based controller was synthesized in^10^ after feedback linearization the nonlinear air-breathing vehicle.

Multirotor aircraft are becoming increasingly popular in both civilian, military, and corporate use. Their applications are vast and include uses in earth science^11^, search and rescue^12^, wild fire suppression^13^, law enforcement^14^, border surveillance^15^, industrial applications^16^, and agriculture^17^. Multirotor aircraft controllers have been designed using robust control^18^ where a hexarotor is designed using a linear quadratic regulator in conjunction with a robust compensator^19^. This controller was designed to be robust against coupling and non-linear dynamics, parametric uncertainties, and external disturbances. Further, trajectory tracking was designed using robust control to decrease sensitivity to unmodeled dynamics and external disturbances^20^.

Despite this extensive research on the application of robust control to aerial vehicles, there has been no exploration of the design space for multirotor vehicles with respect to robustness. Therefore, it is of interest to explore designs for an aerial vehicle which allow it to perform its mission successfully despite the presence of uncertain environmental conditions like wind. Therefore, the main contribution this paper makes is to design the parameters (rather than the control) of the multirotor aircraft to make it robust against external disturbances. In the subsequent sections, the paper presents a systematic treatment of the robust design of such a vehicle (please refer to the appendix for variable definitions).

## Methods

### Conceptual design

The conceptual design is the starting point in systems engineering where a multitude of design configurations are evaluated for performance and robustness following a set of requirements^21^. The current world record for the heaviest payload lifted by a remote controlled multicopter is 134 lb 7.6 oz, according to Guinness World Records^22^. To guarantee a better performance specification, a requirement of a 150 lb payload is set. The weight of the aircraft is generously assumed to be 50 lb for a total of 200 lb. This aircraft shall be able to output this thrust at about 50% power. Providing a margin of 150 lb then brings the maximum thrust output required to 350 lb. Considering current market sizes, a diameter of 0.9 m is set as the maximum diameter of the aircraft sized here.

Muticopters possess greater maneuverability and hovering over conventional single rotor helicopters that allows multirotor vehicles to be relatively simple in design yet highly reliable and maneuverable^23^. Their smaller blades are also advantageous because they possess less kinetic energy, reducing their ability to cause damage. Moreover, higher robustness to turbulence can be achieved by resolution of the reactive moments amongst the rotor frequencies^24^. All two blade electric propellers on the market produced by Advanced Precision Composites (APC) were analyzed to investigate how many propellers and what configuration are needed to produce a thrust of at least 350 lb. Only ringed coaxial propeller configurations are analyzed for the following argument regarding symmetry. In a radially symmetric configuration the deviation in the maximum available angular acceleration about an arbitrary axis at an angle $\gamma$ from the horizontal is minimized. Take for example a quadcopter with an arbitrary angle $\beta$ between the arms and the horizontal. The angular acceleration about an axis is given by (1).

1 $$\begin{aligned} \alpha&= \frac{\tau _\gamma }{J_\gamma } = \frac{\tau _\phi sin\gamma + \tau _\theta cos\gamma }{\frac{1}{2}\left( J_\phi + J_\theta \right) + \frac{1}{2}\left( J_\phi - J_\theta \right) cos(2\gamma )} \nonumber \\&= \frac{K_tL\left( \omega _1^2 + \omega _4^2 - \omega _2^2 - \omega _3^2\right) cos\beta sin\gamma }{4m\left( cos^2\beta cos^2\gamma + sin^2\beta sin^2\gamma \right) } + \frac{K_t\left( \omega _1^2 + \omega _2^2 - \omega _3^2 - \omega _4^2\right) sin\beta cos\gamma }{4m\left( cos^2\beta cos^2\gamma + sin^2\beta sin^2\gamma \right) } \end{aligned}$$

The variation in $\alpha$ over the possible angles $\gamma$ is minimized when $\beta$ equals $45^{\circ }$. This can be generalized to show that the variation in $\alpha$ is minimized for all equiangular configurations (e.g. $60^{\circ }$ between arms for a hexacopter or $45^{\circ }$ between arms for an octocopter). Thus, an equiangular configuration would be more robust to random wind disturbances. A coaxial propeller configuration is chosen as it provides significantly more thrust in a reasonably compact design. A stack of propellers greater than two is not considered due to further increased aerodynamic interference between the propellers^25^. Further, ducting each propeller essentially creates a Kort nozzle which improves efficiency by minimizing tip vortices and by providing additional lift due to the circulation of the flow around the duct^26^. Next, two and four propeller configurations are immediately eliminated as the maximum size propeller at maximum RPM from APC produces insufficient thrust.

**Figure 1:**
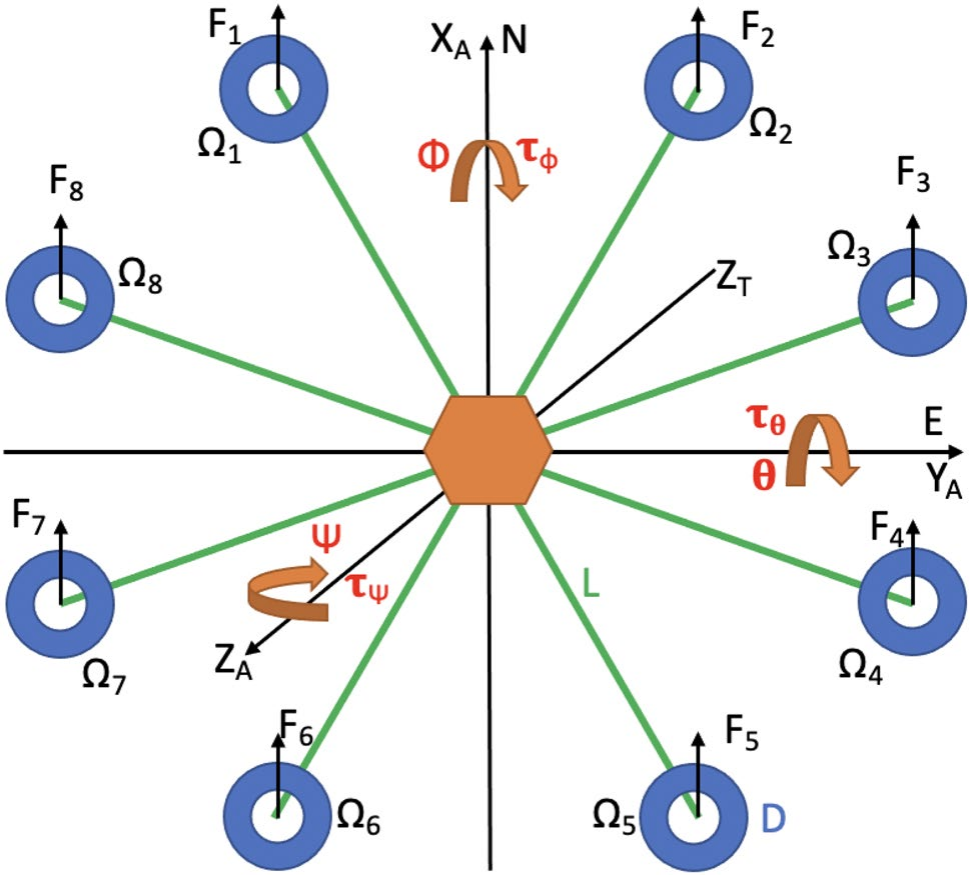
Layout of propeller indexing and orientation with respect to north and east axes at starting position^27^.

All configurations greater than 6 propellers and less than 22 are capable of producing the required thrust. The maximum producible thrust will decrease for numbers of propellers greater than 22 because the maximum allowable propeller size decreases faster than the total thrust produced by having more propellers. For any wind disturbance that creates a pitching or rolling moment, the change in the rotor frequencies will be smaller for a larger number of propellers because the necessary contribution from each propeller will be smaller^24^. The smaller the change in frequencies, the faster the response time will be to reject disturbance and remain in an operational region to maintain stability. Therefore, a decacopter configuration (20 propellers total) would be ideal. However, due to a vastly greater availability of octocopter frames on the market, an octocopter (16 propellers total) configuration is selected. The configuration is illustrated in Fig. 1. An octocopter allows for great redundancy in terms of fault isolation compared to configurations with fewer propellers. An octocopter is able to decompose into each of the previous configurations until a tricopter configuration is reached. Another advantage of coaxial propellers is that the aircraft is able to decompose into an odd number of actuator sets and still maintain yaw stability.

The design parameters considered are the aircraft arm length and propeller size, given the torques are sensitive to changes in these two parameters, see Eqs. (1)–(3) and (25)–(26). The maximum propeller size is limited by what can fit which is given by $D = 2Lsin(22.5^{\circ })$ where *D* is the diameter of the propeller and *L* is the arm length of the aircraft. Finally, combinations of propellers and arm lengths will be optimized under stochastic wind disturbance sequences to determine which design is the most robust. The next step is to develop a model of the system dynamics.

### System modeling

In order to assess a system’s performance and robustness to uncertainties, an underlying model of the system and disturbance dynamics is essential. The octocopter is modeled as a six degree of freedom rigid body consisting of eight arms and sixteen propellers in an octagon configuration. The model relates the position and attitude of the aircraft to the forces and moments produced by the rotors. Each rotor will produce a force given by (2) and a moment given by (3). The propeller frequency $\Omega _i$ represents the frequency for a propeller at a given index. Since there are two propellers at each location, the upper one shall rotate clockwise with this frequency and the lower one shall rotate counterclockwise.

2 $$\begin{aligned} F_i&= K_t\Omega _i^2 \end{aligned}$$

3 $$\begin{aligned} \tau _i&= C_t\Omega _i^2 \end{aligned}$$

The position is described using the north, east, down axis convention where the down axis points into the page in Fig. 1 with respect to the right hand rule. An earth frame and body frame are defined such that the earth frame is an inertial reference frame fixed and oriented to the starting point of the aircraft. The body frame is attached to the aircraft and is aligned with the earth frame at time zero. The attitude of the aircraft is defined using roll, pitch, and yaw angles. Roll is a rotation about the north axis, pitch is a rotation about the east axis, and yaw is a rotation about the down axis where positive is always with respect to the right hand rule. To convert from the body frame to the earth frame, the rotation matrix in (4) is used (with *c*, *s* used to denote sin and cos, respectively)^7^.

4 $$\begin{aligned} {\mathbf{R}} = \left[ \begin{array}{ccc} c\theta c\psi &{}\quad -c\phi s\psi + s\phi s\theta c\psi &{}\quad s\phi s\psi + c\phi s\theta c\psi \\ c\theta s\psi &{}\quad c\phi c\psi + s\phi s\theta s\psi &{}\quad -s\phi c\psi + c\phi s\theta s\psi \\ -s\theta &{}\quad s\phi c\theta &{}\quad c\phi c\theta \end{array}\right] \end{aligned}$$

The translation of the aircraft in the body frame is described by (5).

5 $$\begin{aligned} \left[ \!\begin{array}{ccc} \dot{u} \\ \dot{v} \\ \dot{w} \end{array}\!\right] = -\left[ \!\begin{array}{ccc} P \\ Q \\ R \end{array}\!\right] \times \left[ \!\begin{array}{ccc} u \\ v \\ w \end{array}\!\right] + \frac{1}{m}\left[ \!\begin{array}{ccc} X_A \\ Y_A \\ Z_A + Z_T \end{array}\!\right] + {\mathbf{R}} ^T\left[ \!\begin{array}{ccc} 0 \\ 0 \\ g \end{array}\!\right] \end{aligned}$$

Since *u*, *v*, and *w* are expressed in the body frame, the following Eq. (6) is used to calculate the position in the north, east, down frame of reference.

6 $$\begin{aligned} \left[ \begin{array}{ccc} \dot{P_N} \\ \dot{P_E} \\ \dot{P_D} \end{array}\right] = {\mathbf{R} }\left[ \begin{array}{ccc} u \\ v \\ w \end{array}\right] \end{aligned}$$

The rotation of the aircraft in the earth frame is given by (7).

7 $$\begin{aligned} {\mathbf{J}} \left[ \begin{array}{ccc} \dot{P} \\ \dot{Q} \\ \dot{R} \end{array}\right] = \left[ \begin{array}{ccc} \tau _\phi \\ \tau _\theta \\ \tau _\psi \end{array}\right] - \left[ \begin{array}{ccc} P \\ Q \\ R \end{array}\right] \times {\mathbf{J}} \left[ \begin{array}{ccc} P \\ Q \\ R \end{array}\right] \end{aligned}$$

The moment of inertia matrix, ${\mathbf{J}}$, is defined below.

8 $$\begin{aligned} {\mathbf{J}} = \left[ \begin{array}{ccc} J_\phi &{}\quad 0 &{}\quad 0\\ 0 &{}\quad J_\theta &{}\quad 0\\ 0 &{}\quad 0 &{}\quad J_\psi \end{array}\right] \end{aligned}$$

The moments of inertia for roll and pitch are equal and determined by representing the aircraft as eight thin rods. This results in (9). The moment of inertia for yaw is approximated as twice the moment of inertia for pitch and roll in (10).

9 $$\begin{aligned} J_\phi&= J_\theta = \frac{m}{12}(2L)^2(sin^267.5^{\circ } + sin^222.5^{\circ }) \end{aligned}$$

10 $$\begin{aligned} J_\psi&= 2J_\phi \end{aligned}$$

Finally, the angular body rates are related to the time rate of change of the roll, pitch, and yaw angles by (11), with *c*, *s* used to denote sin and cos, respectively^7^.

11 $$\begin{aligned} \left[ \begin{array}{ccc} P \\ Q \\ R \end{array}\right] = \left[ \begin{array}{ccc} 1 &{}\quad 0 &{}\quad -s\theta \\ 0 &{}\quad c\phi &{}\quad s\phi c\theta \\ 0 &{}\quad -s\phi &{}\quad c\phi c\theta \end{array}\right] \left[ \begin{array}{ccc} \dot{\phi } \\ \dot{\theta } \\ \dot{\psi } \end{array}\right] \end{aligned}$$

The thrust force and the moments are calculated from the rotation rate of the propellers using the allocation matrix given by (12).

12 $$\begin{aligned}&\left[ \begin{array}{cccc} Z_T \\ \tau _\phi \\ \tau _\theta \\ \tau _\psi \end{array}\right] \nonumber \\&\quad = \left[ \begin{array}{cccccccc} -K_t &{} -K_t &{} -K_t &{} -K_t &{} -K_t &{} -K_t &{} -K_t &{} -K_t \\ LK_tc(67.5^{\circ }) &{} -LK_tc(67.5^{\circ }) &{} -LK_tc(22.5^{\circ }) &{} -LK_tc(22.5^{\circ }) &{} -LK_tc(67.5^{\circ }) &{} LK_tc(67.5^{\circ }) &{} LK_tc(22.5^{\circ }) &{} LK_tc(22.5^{\circ })\\ LK_ts(67.5^{\circ }) &{} LK_ts(67.5^{\circ }) &{} LK_ts(22.5^{\circ }) &{} -LK_ts(22.5^{\circ }) &{} -LK_ts(67.5^{\circ }) &{} -LK_ts(67.5^{\circ }) &{} -LK_ts(22.5^{\circ }) &{} LK_ts(22.5^{\circ }) \\ C_t &{} -C_t &{} C_t &{} -C_t &{} C_t &{} -C_t &{} C_t &{} -C_t \end{array}\right] \nonumber \\&\left[ \begin{array}{cccccccc} \Omega _1^2 \\ \Omega _2^2 \\ \Omega _3^2 \\ \Omega _4^2 \\ \Omega _5^2 \\ \Omega _6^2 \\ \Omega _7^2 \\ \Omega _8^2 \end{array}\right] \end{aligned}$$

Next, the model of stochastic wind disturbances acting on the aircraft is developed. The aerodynamic forces are modeled using wind turbulence velocities determined according to the Dryden wind model. (13) gives the aerodynamic forces on the vehicle in the Earth frame basis directions. These forces change as a function of the wind velocities given by the model as well as the rotation and size of the aircraft.

13 $$\begin{aligned} \left[ \!\begin{array}{ccc} X_A \\ Y_A \\ Z_A \end{array}\!\right] = \frac{1}{2}\rho \,C_D \left[ \!\begin{array}{ccc} A_x \\ A_y \\ A_z \end{array}\!\right] \left( {\mathbf{R}} ^T\left[ \!\begin{array}{ccc} u_g \\ v_g + 5 \\ w_g \end{array}\!\right] - \left[ \!\begin{array}{ccc} u \\ v \\ w \end{array}\!\right] \right) ^2 \end{aligned}$$

The areas $A_x$, $A_y$, and $A_z$ are determined by modeling the aircraft as a cylinder using Eqs. (14) and (15)^28^.

14 $$\begin{aligned} A_x = A_y&= 0.708\times 2\pi L\times 0.15 \end{aligned}$$

15 $$\begin{aligned} A_z&= \pi L^2 \end{aligned}$$

The approximate height of the aircraft is 0.15 m. The factor 0.708 gives the component of the wind normal to the aircraft. The effective change in areas due to rolling or pitching is neglected by this model. The Dryden model is effectively a first order filter that converts white noise to colored noise according to Eqs. (16) through (18)^29^.

16 $$\begin{aligned} \dot{u}_g&= -\frac{V}{L_u}u_g + \sigma _u\sqrt{\frac{3V}{L_u}}W_1 \end{aligned}$$

17 $$\begin{aligned} \left[ \begin{array}{c} \dot{v}_g \\ \dot{v}_g^* \end{array}\right]&= \left[ \begin{array}{cc} 0 &{} 1 \\ -\frac{V^2}{L_v^2} &{} -2\frac{V}{L_v}\end{array}\right] \left[ \begin{array}{c} v_g \\ v_g^* \end{array}\right] + \left[ \begin{array}{c} \sigma _v\sqrt{\frac{3V}{L_v}} \\ (1-2\sqrt{3})\sigma _v\sqrt{\left( \frac{V}{L_v}\right) ^3} \end{array}\right] W_2 \end{aligned}$$

18 $$\begin{aligned} \left[ \begin{array}{c} \dot{w}_g \\ \dot{w}_g^* \end{array}\right]&= \left[ \begin{array}{cc} 0 &{} 1 \\ -\frac{V^2}{L_w^2} &{} -2\frac{V}{L_w}\end{array}\right] \left[ \begin{array}{c} w_g \\ w_g^* \end{array}\right] + \left[ \begin{array}{c} \sigma _v\sqrt{\frac{3V}{L_w}} \\ (1-2\sqrt{3})\sigma _w\sqrt{\left( \frac{V}{L_w}\right) ^3} \end{array}\right] W_3 \end{aligned}$$

Here, *V* is the mean wind speed chosen as 5 m/s (in the east direction in addition to the turbulence, without loss of generality). The states $\dot{v}_g^*$ and $\dot{w}_g^*$ are not physically significant and are simply used to calculate the turbulence velocities.

Since the dynamic of the multi-rotor vehicle is unstable, any analysis of the performance of its design requires a stabilizing controller (not necessarily an optimal one) that regulates the system to certain commanded position, which is discussed next.

### Control design

The north, east, and down positions of the aircraft are controlled using PID control. An outer loop takes the desired trajectory in the north and east directions and outputs the desired pitch and roll, given by Eqs. (19) and (20), respectively. An inner loop takes the desired pitch, roll, and height and outputs the desired pitch torque, roll torque, and thrust force given by Eqs. (21), (22), and (23), respectively. Yaw is unaffected by the wind in this model and therefore it is not necessary for it to be controlled. The five controllers represented in Eqs. (19) through (23) have the indicated error values as inputs and the desired forces, torques, or reference angles as outputs.

19 $$\begin{aligned} \theta _{des}&= \left( K_{P,north} + \frac{K_{I,north}}{s} + K_{D,north}s\right) e_{P_N}(s) \end{aligned}$$

20 $$\begin{aligned} \phi _{des}&= \left( K_{P,east} + \frac{K_{I,east}}{s} + K_{D,east}s\right) e_{P_E}(s) \end{aligned}$$

21 $$\begin{aligned} \tau _{\theta ,des}&= \left( K_{P,\theta } + \frac{K_{I,\theta }}{s} + K_{D,\theta }s\right) e_{\theta }(s) \end{aligned}$$

22 $$\begin{aligned} \tau _{\phi ,des}&= \left( K_{P,\phi } + \frac{K_{I,\phi }}{s} + K_{D,\phi }s\right) e_{\phi }(s) \end{aligned}$$

23 $$\begin{aligned} Z_{T,des}&= \left( K_{P,down} + \frac{K_{I,down}}{s} + K_{D,down}s\right) e_{P_D}(s) \end{aligned}$$

The reference for the down direction is set to 0 such that starting from time 0, the aircraft will be in free fall, but the control will return the aircraft to 0. For north and east positions, a trajectory reference is given by the response of the system specified in (24).

24 $$\begin{aligned} P_N^{ref} = P_E^{ref} = {\mathscr {L}}^{-1}\left[ \frac{1}{2s + 1}\right] \end{aligned}$$

A saturation block is added after the pitch and roll controllers to limit the desired angle outputs to ±0.35 radians. This helps keep the aircraft stable and reflects real world limitations of the components. The propellers are also subject to saturation because there exists a maximum angular velocity at which they are rated to rotate. Thus, each propeller is capable of producing a finite force. This limit is given by the manufacturer in (25) for the class of their propellers suitable for multirotors^30^.

25 $$\begin{aligned} \text {RPM}_{max}=145{,}000/D_{in} \end{aligned}$$

Where $D_{in}$ is the diameter of the propeller in inches. To account for this limitation in the simulation, the desired thrust and moments are fed into the allocation matrix given in (12) to calculate each of the desired rotor angular frequencies. These angular frequencies pass through a saturation block and the final force outputs and moments are calculated. The maximum output of the control inputs are a function of the propeller size and the control inputs. This relationship is called constraint tightening. For example, consider the aircraft with zero attitude outputting maximum vertical thrust with all propellers’ frequencies fully saturated. If a command to pitch or roll was given, then the maximum possible vertical thrust would decrease since a difference in propeller frequencies is required to pitch or roll. This behavior is described by (26).

26 $$\begin{aligned} max \left[ \begin{array}{c} Z_T \\ \tau _\phi \\ \tau _\theta \end{array}\right] = f\left( \left[ \begin{array}{c} Z_T \\ \tau _\phi \\ \tau _\theta \end{array}\right] ,\left[ \begin{array}{c} Z_{T,des} \\ \tau _{\phi ,des} \\ \tau _{\theta ,des} \end{array}\right] ,D\right) \end{aligned}$$

Finally, the 8 desired rotor angular velocities are attained by 8 low level PI controllers that manipulate the input voltage and are tuned to have a bandwidth much faster that the outer loops. It is standard practice to use off the shelve electronic speed control (ESC) modules for practical implementation. ESCs can also be used for regenerative braking where the excess mechanical energy while deceleration can be converted to electrical energy used to charge the on-board battery.

In the next section, we present the robust design methodology used to obtain the design point for which the performance of the system consisting of the controllers and the plant model is least sensitive to stochastic wind disturbances.

**Figure 2:**
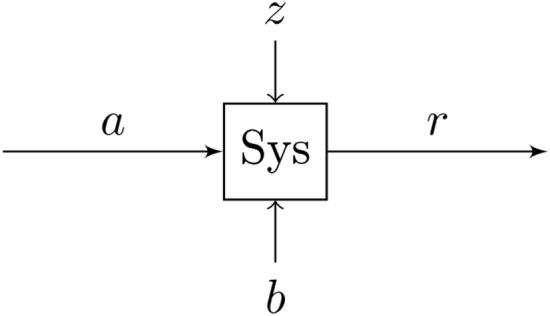
A system at an operating point *a* with a stochastic disturbance *z*. The quality of its response *r* is a function of the parameters given by *b*, as well as the disturbance *z*.

### Robust design

Consider a general dynamical system tracking a reference at an operating point *a* with a stochastic disturbance *z*, resulting in a performance output *r*, see Fig. 2. Suppose that there are variable design parameters given by $b\in {\mathbb {B}}$, the parameter space of the system. ${\mathbb {B}}$ is given by the Cartesian product of the sets of values for the different parameters. We are interested in creating a function $f:{\mathbb {B}}\longrightarrow {\mathbb {R}}^{+},$ where $b \longmapsto f(b)$ whose output provides us with a meaningful measure of how robust the system is to disturbances as well as the quality of its performance.

This is achieved by first developing a cost function that quantifies the error in the system response. Next, due to the stochastic nature of the disturbances introduced, it is necessary to consider the response for many random disturbance sequences and perform some statistical analysis on the data. This insight gives rise to two important robust design techniques, namely, analysis of means, and analysis of variance (ANOM and ANOVA, respectively).

The variance, $\sigma ^2$, of the magnitude of the output error is one good measure of the robustness of the system. A relatively small variance of the error over a wide range of stochastic inputs implies a low sensitivity to the disturbance. Conversely, the mean, $\mu$, of the magnitude of the errors provides a more direct measure of the real performance of the system. A consistently small error will have a small mean, so in addition to providing some insight into the robustness, the mean $\mu$ shows that the performance is good, and not just that it does not change as a function of the disturbance.

The function *f*(*b*), then, is of the form given by (27).

27 $$\begin{aligned} f(b)=\lambda \, \mu _t^2(\text {error}^2(b,t)) + (1-\lambda ) \, \sigma ^2_t(\text {error}^2(b,t)) \end{aligned}$$

for some $\lambda \in [0,1]$, and for the output error of the system as a function of time and the parameters *b*. Note that the means and variances are taken over sufficiently long time horizon. The parameter $\lambda$ must be chosen by the user for the application such that the weights of the mean $\mu$ and the variance $\sigma ^2$ are appropriate for the desired performance.

In general, for a dynamical system with a continuous parameter space ${\mathbb {B}}\subset {\mathbb {R}}^n$ and for a continuous *f*(*b*), it is possible to discern an analytic solution for $b\in {\mathbb {B}}$ that minimizes the cost. However, it would in most cases be a prohibitively difficult problem to solve^31^, and the parameter space for the system in question here is a discrete one. Instead, we perform an analysis of the system for various stochastic sequences called scenarios. This is known as the scenario approach to robust design.

Here, the performance of the resulting controlled system subjected to stochastic wind profiles is evaluated. Scenarios are constructed by passing Gaussian white noise input sequences through the Dryden model and applying those disturbances to the system. To measure the performance of the system in each scenario, the value of the cost function given by (28) is computed.

28 $$\begin{aligned} Cost_{L,D}(i)&= \sum _{k=0}^{K} [P_D({\mathbf{w}} _i(t_0+k\Delta t))]^2 + [1 - P_N({\mathbf{w}} _i(t_0+k\Delta t))]^2 + [1 - P_E({\mathbf{w}} _i(t_0+k\Delta t))]^2 \end{aligned}$$

For a fixed combination of parameters, $(L,D) \in {\mathbb {B}}$, The cost is given by the sum of the squared errors over time. The system is simulated over $K\Delta t$ seconds for each element of ${\mathbb {B}}$. This cost is then a function of the wind profile ${\mathbf{w}} _i(t)$ with index *i* applied to the system. Note that $P_D, P_N,$ and $P_E$ are the outputs for the dynamical system derived in system modeling, subject to the controllers developed in control design. ${\mathbf{w}} _i$ is the wind profile given by the Dryden wind model. They are therefore functions of time, disturbance, and the parameters *L* and *D*.

The mean $\mu$ and the variance $\sigma ^2$ of the cost over the wind profiles ${\mathbf{w}} _i$ can then be calculated for each ordered pair, (*L*, *D*). A new cost function, $f(L,D,\lambda )$ for evaluating robustness of parameters given by (*L*, *D*) can be created that makes use of the mean and variance. The variable $\lambda$ is a user-defined parameter that sets the relative weights assigned to the mean squared and variance terms. In order to be robust, the design must minimize the mean squared and variance of the error by selecting the optimal arm length and propeller pair. The mean squared term ensures that the target position of the aircraft is met with accuracy, whereas the term for variance ensures precision and stability. In order to evaluate the mean squared plus variance, it is necessary for analysis to normalize the values. To perform the normalization, each term in the expression to be minimized is weighted by a factor equal to its minimum when the other term is ignored. The new cost function is given in (29), where $E^{*^2}[Cost_{L,D}({\mathbf{w}} _i)]$ is the minimum value of $E^2[Cost_{L,D}({\mathbf{w}} _i)]$, whereas $Var^*[Cost_{L,D}({\mathbf{w}} _i)]$ is the minimum value of $Var[Cost_{L,D}({\mathbf{w}} _i)]$.

29 $$\begin{aligned} f(L,D,\lambda )&= \lambda \frac{E^2\left[ Cost_{L,D}({\mathbf{w}} _i)\right] }{E^{*^2}\left[ Cost_{L,D}({\mathbf{w}} _i)\right] } + (1-\lambda ) \frac{Var\left[ Cost_{L,D}({\mathbf{w}} _i)\right] }{Var^*\left[ Cost_{L,D}({\mathbf{w}} _i)\right] }\nonumber \\&\text {subject to }L \in [0.2,0.9], \ \lambda \in [0,1],\text { and } D \in \{4,5,6,8,9,11,13,14.5\} \end{aligned}$$

**Figure 3:**
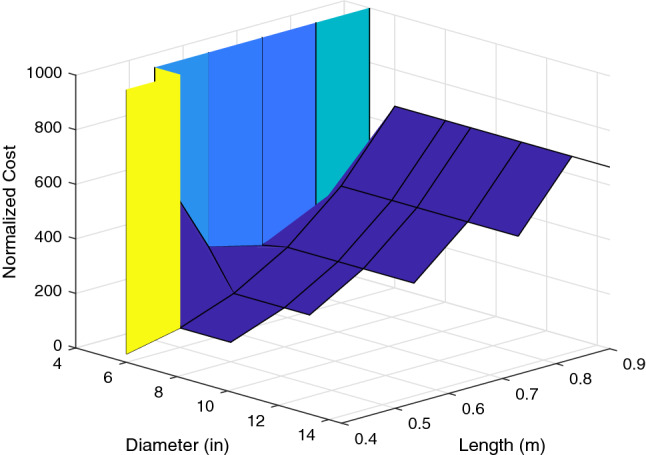
For $\lambda =1$, the cost surface shows the cost as a function of the mean error across the scenarios. Low cost on this surface corresponds to good performance over the varying wind profiles^32^.

**Figure 4:**
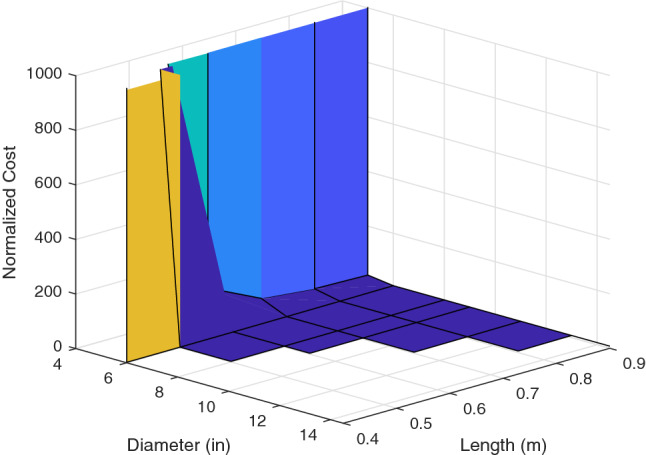
For $\lambda =0$, the cost surface shows the cost as a function of the variance of the error across the scenarios. Low cost on this surface corresponds to robust behavior where the performance is very consistent across the wind profiles^32^.

The value of $\lambda$ is used to weight the importance of the mean versus the variance. For $\lambda =1$, the cost function is based solely on the mean. Conversely, for $\lambda =0$, the cost is a function of only the variance. Figure 3 corresponds to $\lambda =1$, while Fig. 4 corresponds to $\lambda =0$. These surfaces are used to compute the minimum values of the mean and variance terms for normalization of the cost function in (29).

To complete the robust design, we must now minimize this cost function over the viable combinations of parameters given in the parameter space. This minimization is given by (30).

30 $$\begin{aligned} f(L^*,D^*,\lambda )&=\min _{L,D} f(L,D,\lambda )\nonumber \\&\text {such that }\lambda =0.5, \ L \in [0.4,0.9],\; D \in \{4,5,6,8,9,11,13,14.5\},\nonumber \\&\text {where } (L,D) \in {\mathbb {B}}^f\subset L \times D,\; \text {and subject to: }(2){-}(24) \end{aligned}$$

Note that, this process of robust design selects the least sensitive design parameters $b\in {\mathbb {B}}$ that explicitly minimize the mean $\mu _t^2$ and variance $\sigma ^2_t$ of the squared error trajectories $\text {error}^2(b,t)$ with respect to stochastic disturbance trajectories ${\mathbf{w}} _i(t)$ over a sufficiently long time horizon *K*, and by doing so ensures the deviations of the output from the reference remains bounded in spite of disturbances (the notion of robust stability). Taking into account the constraints on the construction and the desired payload developed in the conceptual design, we can eliminate certain combinations of parameters that do not meet the given specifications. The minimization will be performed over the remaining elements of the parameter space. This new viable parameter space, denoted ${\mathbb {B}}^f$, is a subset of the Cartesian product $L \times D$. For this design, we choose $\lambda =0.5$ for a balanced weighting of expectation and variance. The results of this minimization are discussed in the following section.

## Results

Here, we discuss the numerical solution of the minimization problem posed in (30), and the implications of this solution. By applying the maximum angular velocity for each propeller computed using (25) and then computing the maximum resultant thrust using (2), it was determined that no propellers compatible with arm lengths smaller than 0.4 m (smaller than 6 in propellers) would be capable of lifting the payload. We also eliminate pairings of arm lengths with propeller diameters that would cause the propellers to collide based on the symmetric geometry specified in Fig. 1.

**Figure 5:**
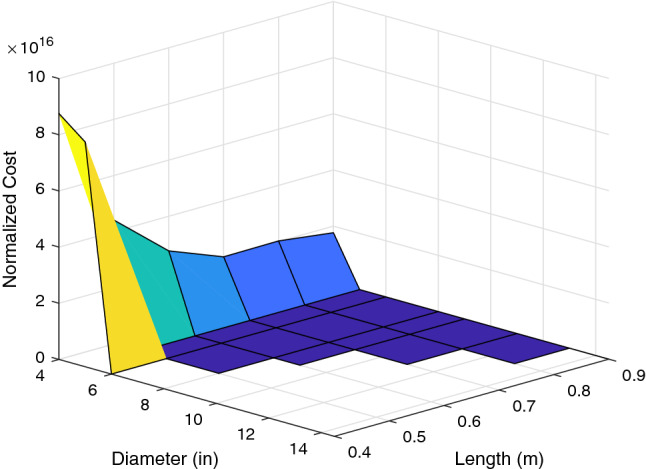
The cost surface shows that for propeller diameters below a certain threshold, the system becomes unstable, resulting in extremely high cost^32^.

**Figure 6:**
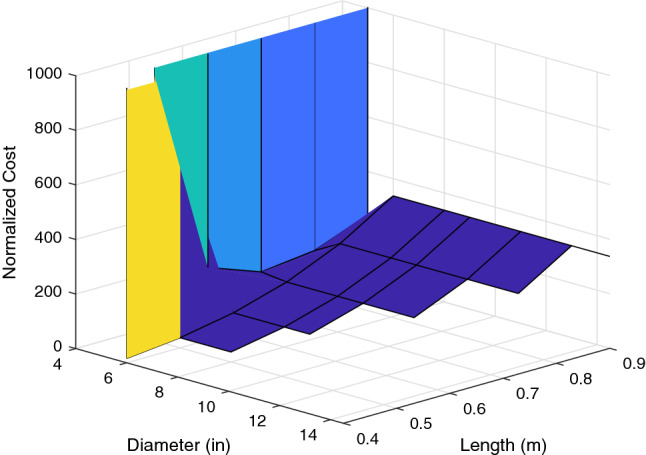
Behavior of the surface for parameters that result in a stable system: this view allows us to pick viable combinations of parameters that will have the best performance^32^.

Because there are two parameters in this system, it is feasible to visualize this cost function for a given $\lambda$ over the parameter space as a surface in ${\mathbb {R}}^3$. There are 8 propellers and 8 arm lengths for a total of 64 combinations in the parameter space. However, 16 of these are eliminated by the thrust requirements because arm lengths than 0.4 m will not be considered. Further, we eliminate 15 more combinations which are not compatible due to over sized propeller dimensions. The surface for $\lambda =0.5$ is shown in Figs. 5 and 6.

**Figure 7:**
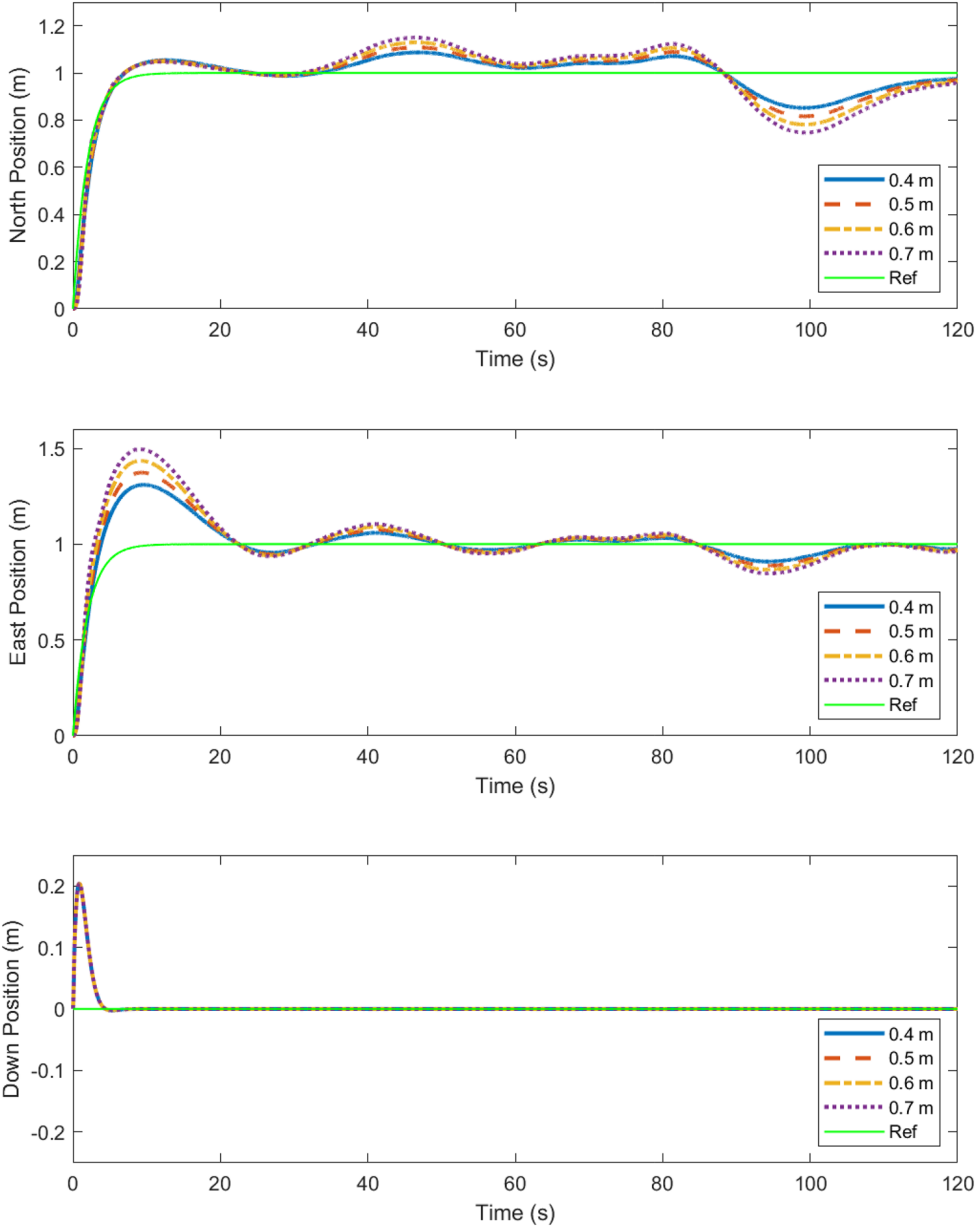
The trajectory of the vehicle is plotted in the N, E, and D directions for combination of arm lengths paired with the largest compatible propeller^32^.

**Figure 8:**
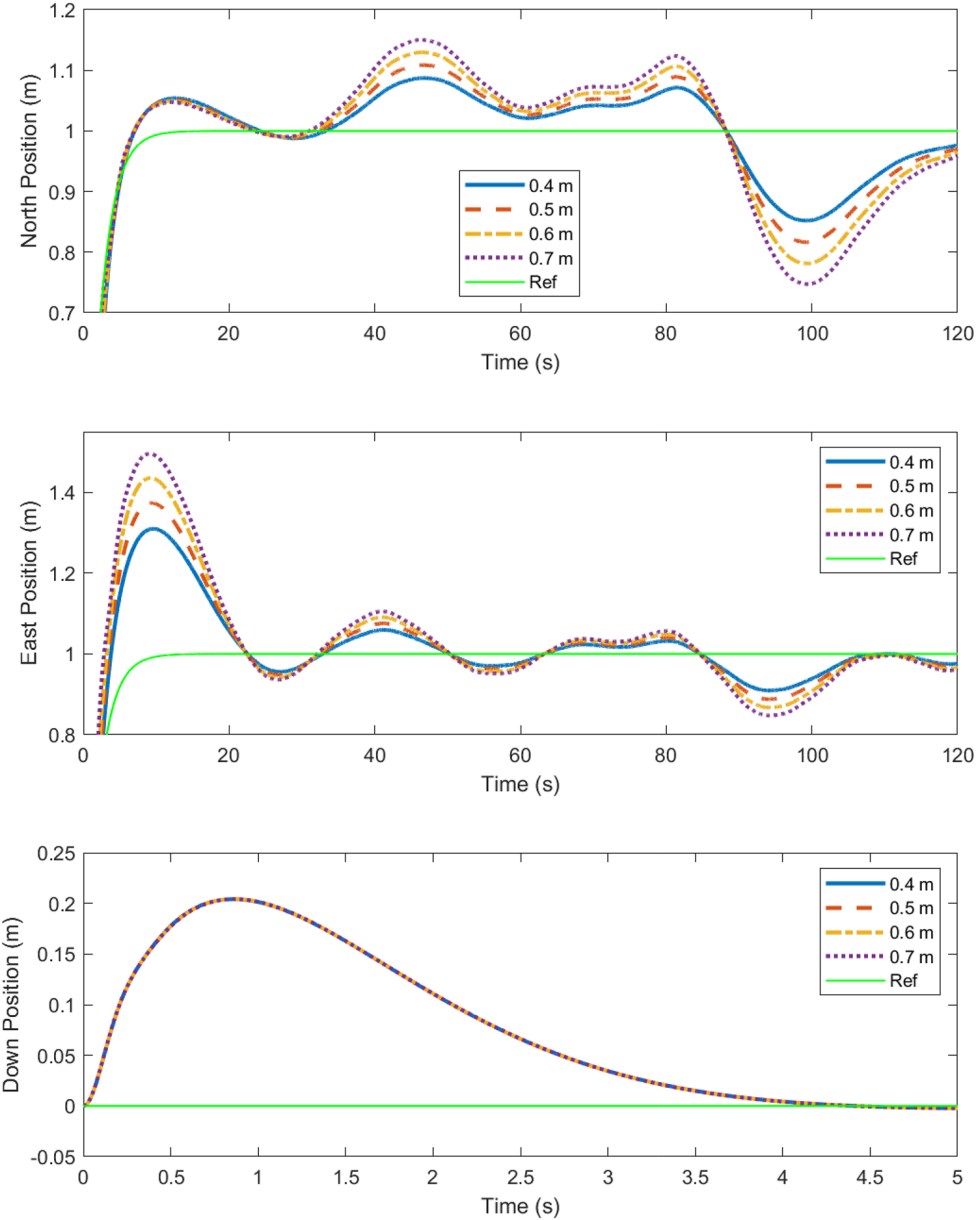
The N, E and D trajectories from Fig. 7 are shown here in greater detail, demonstrating robustness in responses in spite of turbulence due to wind^32^.

The cost surface shows that longer arm lengths perform worse at an exponentially increasing rate. It also shows that for propeller sizes below a certain threshold, the cost is very high. This is because propeller saturation results in bounded thrust. Thus, when the propeller diameter is too small, the system becomes unstable, resulting in a high cost due to the high output error. It is also apparent that for propeller sizes above that threshold, the performance is not affected greatly. This shows that as long as the propeller diameter is sufficient to generate the necessary force, increasing the diameter *D* will not have an immense impact on the robustness of the design, although it will increase the margin for the design. To illustrate the performance implied by the cost surfaces shown, Figs. 7 and 8 show the position of the multirotor in north, east, and down directions compared with the references over the course of a 2 minute scenario.

The trajectories confirm that the shortest arm length combinations perform best, and that the increase in propeller size that comes with the increase in arm length *L* does not affect that trend. Thus, for optimal robustness in this design, 0.4 m arms paired with the largest compatible propellers available must be chosen(in this case, 6 inch APC propellers).

$$\begin{aligned} L^*=0.4\text { m}, \quad D^*=6\text { in} \end{aligned}$$

Because 0.4 m is the smallest arm length that results in a stable system, it is possible for it to be destabilized by an unexpectedly large wind disturbance. In order to ensure stability in higher average wind speeds, a larger diameter to allow for a higher total thrust could be chosen for a larger margin.

## Discussion

In this paper, the requirements for an aerial vehicle were set and a conceptual design was performed. The dynamics of the aircraft were modeled as a six degree of freedom rigid body, and wind was simulated using the Dryden model. PID control loops were added to control the north, east, and down position of the aircraft. Finally, robust design was performed by analyzing a specially designed cost function over a range of different scenarios for all viable arm length and propeller pairs. This cost was defined as a combination of the mean and variance of the sum of the square error in the down, east, and north positions across the scenarios.

It was found that smaller arm lengths produced optimal robustness. However, when propeller saturation was considered, the pairs with propeller diameters below a certain threshold were not viable as they caused instability which resulted in an extremely large cost. It was therefore possible to find an absolute minimum for the cost function over the subset of viable elements of the parameter space. In this case, this occurred for an arm length of 0.4 m with a propeller diameter of 6 in, the largest compatible propeller available.

Note that robust design of the aerial vehicle being an offline process, the inertial measurements are available through model and/or appropriate sensors and the tuned inner and outer loops of the PID controllers suffice to stabilize the multirotor vehicle in each direction. Future contributions to this work could include designing the control system using distributed nonlinear model predictive controllers for 3 dimensional maneuvers^33^ and improving the observation of state variables, in case of unreliable model and/or sensors, by augmenting state estimators^34^. Otherwise, considering more design parameters including motor or battery power constraints and more accurate aerodynamic representations of the propellers could result in minor improvements.

## List of symbols

$A_x$: Aircraft surface area on north face, m$^2$
$A_y$: Aircraft surface area on east face, m$^2$
$A_z$: Aircraft surface area on down face, m$^2$
$C_D$: Coefficient of drag
$C_t$: Propeller coefficient of torque, Nm/RPM$^2$
*D*: Propeller diameter, m
*e*: Error
*F*: Force due to propeller, N
*g*: Acceleration due to gravity, m/s$^2$
$J_\gamma$: Moment of inertia about an axis at an angle $\gamma$ from the horizontal, kg m$^2$
$J_\phi$: Roll moment of inertia, kg m$^2$
$J_\theta$: Pitch moment of inertia, kg m$^2$
$J_\psi$: Yaw moment of inertia, kg m$^2$
$K_t$: Propeller coefficient of force, Nm/RPM$^2$
${\mathbf{J}}$: Moment of inertia matrix
*L*: Aircraft arm length, m
$L_u$: Scale length in u direction, m
$L_v$: Scale length in v direction, m
$L_w$: Scale length in w direction, m
*m*: Mass of aircraft, kg
*P*: Roll angular body rate, rad/s
$P_N$: North position of aircraft in earth frame, m
$P_E$: East position of aircraft in earth frame, m
$P_D$: Down position of aircraft in earth frame, m
*Q*: Pitch angular body rate, rad/s
*R*: Yaw angular body rate, rad/s
${\mathbf{R}}$: Body to earth rotation matrix
*t*: Time, s
*u*: North velocity of aircraft expressed in body frame, m/s
$u_g$: North turbulence velocity in earth frame, m/s
*V*: Mean wind speed, m/s
*v*: East velocity of aircraft expressed in body frame, m/s
$v_g$: East turbulence velocity in earth frame, m/s
$v_g^*$: State variable to solve $v_g$, m/s
*W*: Gaussian white noise random variable
*w*: Down velocity of aircraft expressed in body frame, m/s
$w_g$: Down turbulence velocity in earth frame, m/s
$w_g^*$: State variable to solve $w_g$, m/s
$X_A$: North aerodynamic force, N
$Y_A$: East aerodynamic force, N
$Z_A$: Down aerodynamic force, N
$Z_T$: Total vertical thrust, N
$$\alpha $$: Angular acceleration, m/s$$^2$$
$\beta$: Angle between aircraft arms and horizontal, rad
$\gamma$: Angle between an arbitrary axis and the horizontal, rad
$\theta$: Pitch angle, rad
$\rho$: Density of air, kg/m$^3$
$\sigma _u$: Turbulence intensity in direction of u
$\sigma _v$: Turbulence intensity in direction of v
$\sigma _w$: Turbulence intensity in direction of w
$\tau _\gamma$: Torque about an axis at an angle gamma from the horizontal, Nm
$\tau _\theta$: Pitch torque, Nm
$\tau _\phi$: Roll torque, Nm
$\tau _\psi$: Yaw torque, Nm
$\phi$: Roll angle, rad
$\psi$: Yaw angle, rad
$\Omega$: Coaxial propeller set angular velocity, RPM
$\omega$: Quadrotor propeller angular velocity, RPM

## Subscripts

*i*: Index
*des*: Desired

## References

[1] Park G-J, Lee T-H, Lee KH, Hwang K-H (2006). Robust design: an overview. AIAA J..

[2] Jingjing Z (2019). Six sigma robust design optimization for thermal protection system of hypersonic vehicles based on successive response surface method. Chin. J. Aeronaut..

[3] Arvidsson M, Gremyr I (2008). Principles of robust design methodology. Qual. Reliab. Eng. Int..

[4] Meng, W. & Ma, D. Robust design optimization method for aircraft. *2010 3rd International Symposium on Systems and Control in Aeronautics and Astronautics* 430–434 (2010).

[5] Padulo M, Guenov MD, Forth SA (2008). Robust aircraft conceptual design using automatic differentiation in Matlab. Adv. Autom. Differ. Lect. Notes Comput. Sci. Eng..

[6] Dutta, A. *Design and Certification of Industrial Predictive Controllers* (Ghent University, 2014).

[7] Xu, J., Shi, P., Lim, C.-C., Cai, C. & Zou, Y. Reliable tracking control for under-actuated quadrotors with wind disturbances. *IEEE Trans. Syst. Man Cybern. Syst.* (2018).

[8] Xu J, Shi P, Lim CC, Cai C, Zou Y (2016). Integrated structural parameter and robust controller design for attitude tracking maneuvers. IEEE/ASME Trans. Mechatron..

[9] Hentabli, K., Akhrif, O. & Saydy, L. Robust longitudinal flight control system under weight and center of gravity uncertainty. In *CCECE 2003-Canadian Conference on Electrical and Computer Engineering. Toward a Caring and Humane Technology (Cat. No. 03CH37436)*, vol. 3, 1743–1748 (IEEE, 2003).

[10] Ur Rehman, O., Petersen, I. R. & Fidan, B. Feedback linearization-based robust nonlinear control design for hypersonic flight vehicles. *Proc. Inst. Mech. Eng. Part I J. Syst. Control Eng.* **227**, 3–11 (2013).

[11] Fladeland M (2011). The Nasa sierra science demonstration programme and the role of small-medium unmanned aircraft for earth science investigations. Geocarto Int..

[12] Silvagni M, Tonoli A, Zenerino E, Chiaberge M (2017). Multipurpose UAV for search and rescue operations in mountain avalanche events. Geomat. Nat. Hazards Risk.

[13] Bushnaq, O. M., Chaaban, A. & Al-Naffouri, T. Y. The role of UAV-IoT networks in future wildfire detection. *IEEE Internet Things J.* (2021).

[14] Constantinescu, S.-G. & Nedelcut, F. UAV systems in support of law enforcement forces. In *International Conference of Scientific Paper AFASES*, vol. 2011, 1211–1219 (2011).

[15] Vijayanandh, R., Kumar, J. D., Kumar, M. S., Bharathy, L. A. & Kumar, G. R. Design and fabrication of solar powered unmanned aerial vehicle for border surveillance. In *Proceedings of International Conference on Remote Sensing for Disaster Management*, 61–71 (Springer, 2019).

[16] Guerra E, Munguía R, Grau A (2018). UAV visual and laser sensors fusion for detection and positioning in industrial applications. Sensors.

[17] Radoglou-Grammatikis P, Sarigiannidis P, Lagkas T, Moscholios I (2020). A compilation of UAV applications for precision agriculture. Comput. Netw..

[18] Santoso F, Garratt MA, Anavatti SG, Petersen I (2018). Robust hybrid nonlinear control systems for the dynamics of a quadcopter drone. IEEE Trans. Syst. Man Cybern. Syst..

[19] Liu H, Derawi D, Kim J, Zhong Y (2013). Robust optimal attitude control of hexarotor robotic vehicles. Nonlinear Dyn..

[20] Li Z, Ma X, Li Y (2020). Robust trajectory tracking control for a quadrotor subject to disturbances and model uncertainties. Int. J. Syst. Sci..

[21] Van Nguyen N, Lee J-W, Lee Y-D, Park H-U (2014). A multidisciplinary robust optimisation framework for UAV conceptual design. Aeronaut. J..

[22] Swatman, R. Video: Giant multicopter drone that could lift weight of a human adult flies into record books (2016). http://www.guinnessworldrecords.com/news/2016/1/video-giant-multicopter-drone-that-could-lift-weight-of-a-human-adult-flies-into-413032

[23] Michieletto G, Ryll M, Franchi A (2018). Fundamental actuation properties of multirotors: Force-moment decoupling and fail-safe robustness. IEEE Trans. Robot..

[24] Toledo J, Acosta L, Perea D, Morales N (2015). Stability and performance analysis of unmanned aerial vehicles: Quadrotor against hexrotor. IET Control Theory Appl..

[25] Coleman, C. A survey of theoretical and experimental coaxial rotor aerodynamic research. *NASA-TP-3675* (1997).

[26] Rutkowski, M. & Krusz, W. Design and analysis of ducted fan for multi-rotor VTOL UAV. *PhD Interdisciplinary Journal Politechnike Gdanska*, 149–155 (2013).

[27] Powerpoint. Version 16.43. *Microsoft Corporation* (2020).

[28] Solovyev V, Finaev V, Zargaryan Y, Shapovalov I, Beloglazov D (2015). Simulation of wind effect on a quadrotor flight. ARPN J. Eng. Appl. Sci..

[29] Tran, N. K. *Modeling and control of a quadrotor in a wind field* (McGill University, 2015) (**Unpublished Master’s Thesis)**.

[30] APC. RPM limits for aircraft propellers. https://www.apcprop.com/technical-information/rpm-limits/ (2021).

[31] Alyaqout SF, Papalambros PY, Ulsoy AG (2011). Combined robust design and robust control of an electric dc motor. IEEE/ASME Trans. Mechatron..

[32] MATLAB. Version 9.4. *The MathWorks Inc.* (2018).

[33] Dutta A, Ionescu C, De Keyser R (2015). A pragmatic approach to distributed nonlinear model predictive control: Application to a hydrostatic drivetrain. Optim. Control Appl. Methods.

[34] Dutta A, Langbort C (2017). Confiscating flight control system by stealthy output injection attack. J. Aerosp. Inf. Syst..

